# Supplementary material for: A Homogeneous Colorimetric Strategy Based on Rose-like CuS@Prussian Blue/Pt for Detection of Dopamine
Source: Sensors (Basel). 2023 Nov 7;23(22):9029. doi: 10.3390/s23229029 (PMC10675490; doi:10.3390/s23229029)
Supplement: Supplementary file 1 [file sensors-23-09029-s001.zip › sensors-2596855-supplementary.pdf]

Supplementary Information

A Homogeneous Colorimetric Strategy Based on Rose-Like CuS@Prussian Blue/Pt for Detection of Dopamine

Di Yang, Jiao Ran, Huafei Yi, Pujin Feng and Bingqian Liu *

Guizhou Engineering Laboratory for Synthetic Drugs (Ministry of Education of Guizhou Province), College of Pharmacy, Guizhou University, Guiyang 550025, China.

Reagents and Apparatus

Iron trichloride hexahydrate ($FeCl_3{\cdot}6H_2O$, AR), Sodium borohydride, and Anhydrous calcium chloride were purchased from Sinopharm Chemical Reagent Co., Ltd. (Shanghai, China); Thiourea, Dopamine hydrochloride ($C_8H_{12}C_lNO_2$, AR), L-ascorbic acid ($C_6H_8O_6$), and Polyethylene glycol (PEG) were purchased from Macklin Reagent Co., Ltd.; Copper chloride dihydrate ($CuCl_2{\cdot}2H_2O$, AR) was purchased from Yongda Chemical Reagent Co., Ltd. (Tianjin, China); Glycine ($C_2H_5NO_2$) was purchased from Solaibao Technology Co. Ltd. (Beijing, China); Potassium ferrocyanide ($K_4[Fe(CN)_6]$), Sodium chloride, Glucose ($C_6H_{12}O_6$), Potassium chloride, and Hydrogen peroxide (H_2O_2 ,30%) were purchased from Jinshan Chemical Reagent Co. Ltd. (Chengdu, China)； O-phenylene diamine ($C_6H_8N_2$, OPD) was purchased from Aladdin Biochemical Technology Co., Ltd. (Shanghai, China); 2,2' -bisazo-3-ethylbenzothiazoline 6-sulfonic acid ($C_{18}H_{24}N_6O_6S_4$, ABTS) was purchased from Sangon Bioengineering Co., Ltd. (Shanghai, China); Polyvinylpyrrolidone (PVP), L-glutamate ($C_5H_9NO_4$), 3,3',5,5' -tetramethylbenzidine ($C_{16}H_{20}N_2$, AR) were purchased from Yuanye Biological Co., Ltd. (Shanghai, China); Absolute ethanol (C_2H_6O),Sodium hydroxide (NaOH, AR),Disodium hydrogen phosphate (Na_2HPO_4),and Potassium dihydrogen phosphate (NaH_2PO_4) were purchased from Kemio Chemical Reagent Co., Ltd. (Tianjin, China).

Apparatus

Scanning electron microscope (SEM) was implemented on the JEOL 7600 (JEOL, Tokyo, Japan). X-ray photon spectroscopy (XPS) was tested on Thermo Fisher

Scientific K-Alpha (Waltham, MA, USA). Energy dispersive spectroscopy (EDS) and element mapping were acquired from Oxford X-MAX (Zeiss, Oberkochen, Germany) and Zeiss Sigma 300 (Zeiss, Oberkochen, Germany), respectively. The UV-vis absorption spectra were tested on a UV-5500PC UV-visible spectrophotometer (Metash, Shanghai, China).

Synthesis of CUS, CuS@PB, and CuS@PB/pt

All of the following materials have been prepared based on literature with slight modifications. Firstly, 0.684 g $CuCl_2 \cdot 2H_2O$, 1.216 g CH_4N_2S, and 1.6 g PEG were added into three beakers containing 20 mL of distilled water, respectively. The three solutions are called A, B, and C, respectively. Afterwards, the blue uniform solution A and colorless uniform solutions B and C were obtained by magnetic stirring for 5 min. Subsequently, solution A and solution C were mixed for 10 min, followed by the slow addition of solution B under continuous stirring. The white emulsion was obtained. Then the white emulsion was shifted into a reactor with a polytetrafluoroethylene lining, which was put in a drying cabinet at 150 °C for 10 h. After the reactor is cooled, the obtained precipitate is washed with water and alcohol alternately five times. Finally, black powder was obtained by drying the washed precipitate in a drying cabinet.

The synthesis process of CuS@PB NPs was based on Li's methods [1] and modified slightly. CuS@PB NPs were prepared via a hydrothermal method previously. Core-shell CuS@PB NPs were prepared through an in situ synthesis method. In a typical synthesis, CuS NPs (50 mg), $FeCl_3$ (0.05 mM), and CA (0.25 mM) were dissolved in ultrapure water (10 mL). Then, an aqueous solution (10 mL) containing $K_4[Fe(CN)_6]$ (0.05 mM) and CA (0.25 mM) was dropped slowly into the above solution with vigorous stirring at room temperature. A blue-green colloidal solution was obtained by incubating the reaction mixture in a water bath at 40 °C with continuous stirring for 30 min. Finally, the core-shell CuS@PB NPs were collected from the suspension by centrifugation. Then, it was washed with acetone and ultrapure water three times. CuS@PB/Pt was synthesized according to a previously published protocol, but with

slight modifications. 20 mg of CuS@PB and 0.01 g of PVP-K30 were added to 8 mL of ethanol under magnetic stirring for 30 min. Then, 10 mL of 1 mM $H_2PtCl_6 \cdot 6H_2O$ aqueous solution was added to the mixture and stirred for an additional 10 min. Subsequently, the mixture was mixed with 0.5 mL of 50 mM $NaBH_4$ aqueous solution, stirred for 30 min, and centrifuged at 6708×g for 10 min to obtain CuS@PB/Pt.

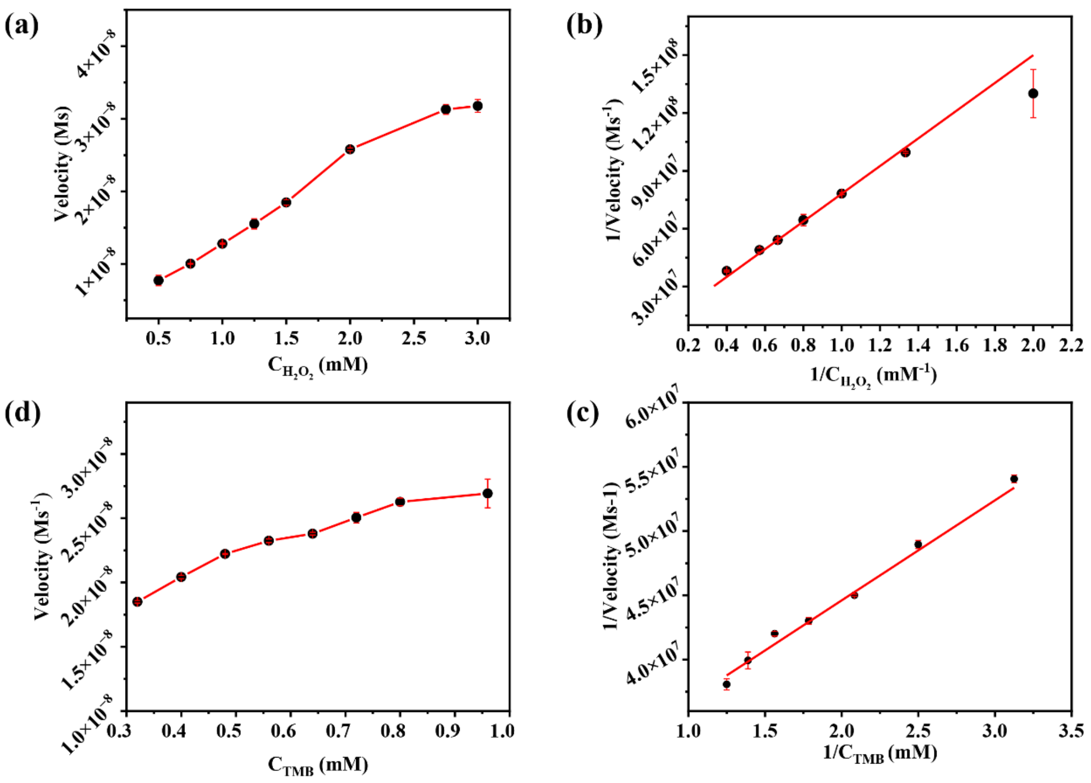

Figure S1. (a,c) Steady-state kinetic analysis utilizing the Michaelis–Menten model of H_2O_2 and TMB; (b,d) Lineweaver–Burk double-reciprocal model of H_2O_2 and TMB.

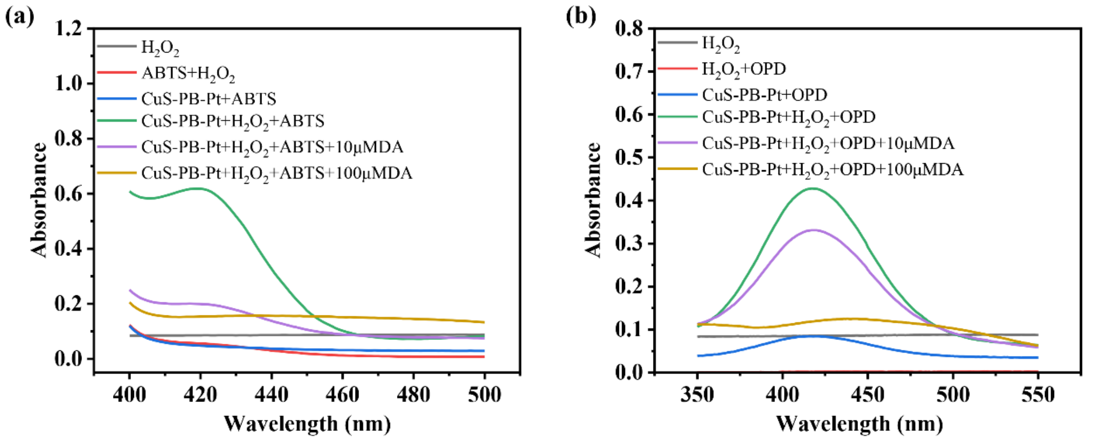

Figure S2. UV-vis absorption spectra with different chromogenic reagents of the addition of DA at different concentrations ((a)ABTS; (b) (OPD))

Table S1. Determination of dopamine in human serum.

Added DA (μM)	Method	Measured (mean ± SD，μM，n=3)	Recovery (%)
1	RGB	0.88±0.11	88.71
	UV	0.94±0.23	94.18
	LC-MS/MS	1.12±0.11	112.03
2	RGB	2.13±0.09	106.62
	UV	2.11±0.03	105.55
	LC-MS/MS	1.88±0.15	94.25
5	RGB	5.00±0.15	100.09
	UV	4.72±0.09	94.39
	LC-MS/MS	5.03±0.21	100.5
8	RGB	8.28±0.05	103.51
	UV	8.14±0.27	101.75
	LC-MS/MS	8.12±0.07	101.51
10	RGB	10.76±0.39	107.58
	UV	10.27±0.29	102.71
	LC-MS/MS	10.9±0.19	109.09
30	RGB	/	/
	UV	29.02±1.29	96.74
	LC-MS/MS	29.62±0.38	98.73

References

1. Li, L. H.; Zhang, P.; Li, Z. Y.; Li, D. Y.; Han, B.; Tu, L.; Li, B.; Wang, Y. G.; Ren, L.; Yang, P. Y.; Ke, S. K.; Ye, S. F.; Shi, W., CuS/Prussian blue core-shell nanohybrid as an electrochemical sensor for ascorbic acid detection. *Nanotechnology* **2019,** 30, (32),1-12.

2. Pi, Z. F.; Wang, Q. Q.; Zhang, J.; Song, F. R.; Liu, Z. Q., Effect of Schisandra Fruit on Neurochemicals in Hippocampus of Diabetic Encephalopathy Rat Using Online MD-HPLC-MS/MS. *Chemical Journal of Chinese Universities-Chinese* **2015,** 36, (3), 442-448.

3. Yan, M.; Ge, S. G.; Lu, J. J.; Yu, J. H., Fluorescence Quenching Method for Determination of Dopamine Based on Double Molecular Recognition. *Chinese Journal of Analytical Chemistry* **2011,** 39, (11), 1711-1715.

4. Gong, Q.-j.; Han, H.-x.; Wang, Y.-d.; Yao, C.-z.; Yang, H.-y.; Qiao, J.-l., An electrochemical sensor for dopamine detection using poly-tryptophan composited graphene on glassy carbon as the electrode. *New Carbon Materials* **2020,** 35, (1), 34-41.
5. Liu, S. M.; Zhou, X. Y.; Lv, C. Y.; Liu, R.; Li, S. Y.; Yang, G. Y., A novel bromelain-MnO2 biosensor for colorimetric determination of dopamine. *New Journal of Chemistry* **2021,** 45, (1), 92-97.
